# Green Gentrification and Health: A Scoping Review

**DOI:** 10.3390/ijerph18030907

**Published:** 2021-01-21

**Authors:** Na’Taki Osborne Jelks, Viniece Jennings, Alessandro Rigolon

**Affiliations:** 1 Environmental and Health Sciences Program, Spelman College, Atlanta, GA 30314, USA; 2 West Atlanta Watershed Alliance, Atlanta, GA 30310, USA; 3 Department of Public Health, Agnes Scott College, Decatur, GA 30030, USA; vjennings@agnesscott.edu; 4 Department of City and Metropolitan Planning, The University of Utah, Salt Lake City, UT 84112, USA; alessandro.rigolon@utah.edu

**Keywords:** green gentrification, green space, parks, public health

## Abstract

Urban greening initiatives are often linked to enhanced human health and wellbeing, but they can also be a driver of gentrification. To date, few studies have focused on how green gentrification shapes health. In this scoping review, we analyzed existing peer-reviewed research on how greening initiatives in gentrifying neighborhoods impact health, well-being, and health pathways (e.g., physical activity, affordable housing). Using a multi-step approach to scoping the literature (including searches in PubMed, JSTOR, and Google Scholar), we identified 15 empirical studies that met our inclusion criteria. We found studies focusing on green space use, physical activity, sense of community, safety, and self-reported health. Overall, longtime, marginalized residents are negatively impacted by green gentrification as they experience a lower sense of community, feel that they do not belong in green space, and, in many studies, use green space less often than newcomers. Overall, the research in this area is limited, and more studies on mental health and cardiovascular health markers could advance this literature. Based on the limited available evidence, we suggest that public health, urban planning, and parks professionals could collaborate to enhance the use of green space for marginalized residents and their feelings of inclusion in gentrifying areas.

## 1. Introduction

Gentrification is an ongoing challenge for many cities worldwide [1]. It is often described as the process by which under-resourced neighborhoods are developed and experience a migration of affluent newcomers [2,3]. Specifically, gentrification has been defined as a sociocultural phenomenon in which market forces contribute to the renovation of neighborhoods that have seen decades of disinvestment [4,5]. In some cases, however, the significant increases in housing prices resulting from gentrification contribute to the displacement of low-income residents, especially renters, who can no longer afford to live in rapidly gentrifying neighborhoods [6].

Gentrification is a concept made up of multiple dimensions [2], and the way it manifests in cities can vary. For example, one United States (U.S.) study showed that the nation’s largest cities experience gentrification at a greater intensity than other cities, but gentrification can also occur in smaller cities when in conjunction with the improvement of central business districts [4]. Entities such as the National Community Investment Coalition have recently seen increased concerns about gentrification and displacement among its membership, which spans over six hundred local and national non-profit, government, and educational organizations in the U.S. [4]. According to the Centers for Disease Control and Prevention (CDC), gentrification is a “housing, economic, and health issue that affects a community’s history and culture and reduces social capital, and it ‘has the potential to cause displacement of long-time residents and businesses’” [7].

Three systematic reviews recently examined the research connecting gentrification and human health [8,9,10]. The authors of these reviews report that gentrification can impact a broad variety of health outcomes, ranging from physical to mental health, and that the directions of associations between gentrification and health are not consistent [8,9]. Yet, two reviews found that gentrification has worse health impacts for marginalized groups, such as Black, low-income, and elderly people, than for more privileged groups [8,10]. These results can be explained, at least in part, by what we know about the connections between housing and health. Housing is considered a social determinant of health, and specifically, housing affordability, stability, quality, and safety affect multiple dimensions of health and well-being [11,12]. In gentrifying neighborhoods, the availability of affordable housing is gradually shrinking [13], which can potentially impact the health and well-being of the most marginalized residents.

Among the different mechanisms that trigger gentrification, recent research has paid particular attention to greening initiatives, such as investments in parks, greenways, trees, and environmental remediation efforts [14,15,16,17,18]. Specifically, scholars have used the term ‘green gentrification,’ or ‘environmental gentrification,’ to define the influx of wealthier new residents to previously low-income neighborhoods where some greening initiatives are implemented [14,16,17,18]. More recently, the concept of ‘green climate gentrification’ or ‘climate gentrification’ has been coined to describe the arrival of new affluent residents to low-income neighborhoods, due in part to greening efforts to bolster climate resilience [19]. Green gentrification has been documented in cities around the world, including in the U.S., Spain, Belgium, and South Korea [14,15,17,20]. When focusing on the creation of new parks, research has shown that green gentrification is more likely to occur when parks are established near downtowns or designed as greenways, including active transportation trails [14,17]. Green gentrification is a particularly insidious process because, in many circumstances, greening initiatives are motivated by health equity goals, such as improving the health and well-being of marginalized groups [21,22].

In the last few years, scholars and activists have expressed a growing interest in the links between green gentrification and health, or more broadly, whether and how green space is associated with health in neighborhoods that are gentrifying [21,23]. Although there have been at least three systematic reviews focusing on how gentrification (in general) impacts human health [8,9,10], to our knowledge, no review has focused specifically on the connections between green gentrification and health. We argue that examining whether green gentrification improves or aggravates the health of marginalized groups is key to informing urban greening initiatives in low-income and racially/ethnically diverse neighborhoods that are susceptible to gentrification. In other words, urban planners, public health professionals, and elected officials could benefit by knowing whether marginalized groups such as Black, Indigenous, and People of Color (BIPOC) communities experience a net gain in their health and well-being when green gentrification occurs. On one hand, marginalized residents in neighborhoods experiencing green gentrification gain new access to high-quality green spaces, with potential benefits related to physical activity, socialization, and ecosystem services [24,25]. Conversely, rents likely increase, residents might not feel welcome in the new green spaces, or worse, they might be forced to relocate due to excessive increases in housing prices, presumably resulting in a loss of social networks [21,26]. Researchers note that the paradox and consequences of green gentrification can lead to a shift in the ecosystem services that are accessible to marginalized communities [27]. Thus, these trade-offs call for a review of the latest evidence on the links between green gentrification and health.

Analyzing existing peer-reviewed research, we sought to answer the following question: How does green gentrification influence human health and pathways to health? We conducted a scoping review to synthesize existing evidence and to assess the scope of literature on the relationship between green gentrification and health outcomes and/or pathways. We also explored the health effects of green space in neighborhoods that are gentrifying, regardless of whether the green space, itself, was a driver of gentrification. This is the first review article, to our knowledge, that has explored the health implications of green gentrification. As noted earlier, this is an important knowledge gap to bridge since greening initiatives in marginalized communities may spur gentrification despite an initial goal to support health equity [21,22]. Based on the findings of this review, we present broad recommendations for urban planners, public health professionals, and others working on urban greening projects. We also discuss existing gaps and future research needs in the context of green gentrification and health.

## 2. Methods

**Figure 1:**
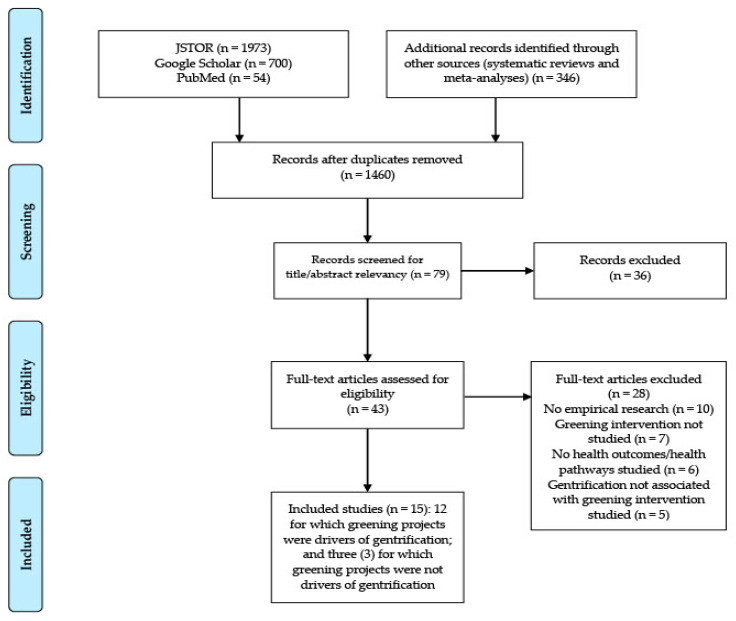
PRISMA (Preferred Reporting Items for Systematic Reviews and Meta-Analyses) Flow Diagram.

We used a multi-step, multi-method approach to scope the literature on green gentrification and health. Our approach builds on Preferred Reporting Items for Systematic reviews and Meta-Analyses extension for Scoping Reviews (PRISMA-ScR) guidelines [28] (see Table S1 in the Supplementary Materials), as well as other guidance [29] and precedents used in previous scoping reviews [29,30]. Given the relative novelty of this body of research, we believe our multi-step, multi-method approach was needed to capture the range of studies on green gentrification and health (see Figure 1).

### 2.1. Inclusion Criteria

We included peer-reviewed articles that investigated the linkages between green gentrification and human health along with pathways in this relationship and published any time before 19 November 2020. Specifically, articles that met the following criteria were included:

- Examine the linkages between green gentrification (or related concepts such as climate gentrification) and health outcomes (physical or mental), well-being, or health pathways. Such pathways represent mechanisms through which green space can promote human health, including reducing harm (e.g., exposure to air pollution), restoring capacities (e.g., stress relief), and building capacities (e.g., physical activity promotion) [31]; OR
- Study the linkages between green space and health outcomes, well-being, or health pathways in neighborhoods undergoing gentrification; AND
- Analyze such linkages either quantitatively or qualitatively (thus, excluding reviews); AND
- Be published in English

Articles with study populations throughout the world were eligible for inclusion. Due to inconsistent language for green-induced gentrification, we considered as eligible a range of terms (e.g., green gentrification, environmental gentrification, ecological gentrification, green climate gentrification, and climate gentrification). The most common reasons for exclusion at the final stage of evaluation (*n* = 43 to *n* = 15) were that the articles addressed gentrification without referencing green-induced gentrification or that articles focused on green gentrification but did not consider health, well-being, or health pathways.

### 2.2. Search Strategies and Study Selection

To identify the articles meeting the inclusion criteria, we undertook three steps. First, we initially conducted literature searches in PubMed, JSTOR, and Google Scholar using search terms describing green gentrification (and related terms such as environmental and ecological gentrification), green spaces, health, and health pathways (see Table S2 in the Supplementary Materials). We determined the final search expressions after trialing the searches and, specifically, added ‘green climate gentrification’ and ‘climate gentrification’ after reviewing a few abstracts from initial searches. We used this approach because scholars have used a variety of terms to name gentrification induced by greening initiatives. For PubMed and JSTOR, we screened all articles resulting from each search (see Figure 1). Given a large number of results in Google Scholar (more than 100,000 entries, including gray literature), we only screened the first 100 articles from each search (see Table S2 in the Supplementary Materials), following an approach cited in the literature [32].

These searches resulted in a total of 1460 articles; after abstract screening, we identified 40 articles; and after full-text screening, we determined that 12 articles met the inclusion criteria. After independent evaluations, all authors reached an agreement about whether each article under consideration should be included in the review. We imported records into EndNote (Clarivate Analytics, Beijing, China) for coding and management.

Second, from the above searches, we identified five systematic reviews that focused on either gentrification and health [8,9,10,33] or ways to limit environmental gentrification [34]. Following the example of previous scoping reviews [29], we screened their references to identify additional relevant articles that met the inclusion criteria. Similar to another scoping review on green spaces and health [29], we included additional references identified in key review articles. Through this process, we found two additional articles to include in our review.

Third, based on the precedent of other scoping reviews [30], we reviewed the references of the 14 included articles identified through steps 1 and 2 (12 and two papers, respectively). Through such a review, we identified one additional article that met the inclusion criteria. Thus, our three-step search process resulted in 15 articles that we included in our scoping review.

### 2.3. Data Extraction and Synthesis

Relevant information was gathered from the 15 included articles identified in the scoping review. Specifically, we extracted key details from the articles (e.g., study methodology, location, study population, health outcomes or pathways examined, summary of findings) which are outlined in Table 1.

Given the limited number of included studies and the notable heterogeneity among them, we did not seek to quantify trends regarding positive or negative health effects from green gentrification or green space in gentrifying neighborhoods. Rather, we sought to synthesize the available evidence qualitatively, specifically comparing study findings that focused on similar greening interventions (e.g., parks or greenways) and similar health outcomes or pathways. We also distinguished between studies in which greening was one of the engines of gentrification and where it was not.

## 3. Results

### 3.1. Study Characteristics

Through our search process, we identified 15 articles that met our inclusion criteria, published from 2012 to 2020. More than two-thirds (11 of 15) of these studies were published in the last three years (2017–2020). We summarized the main characteristics of each study in Table 1. The methodologies used were almost evenly distributed among the 15 articles: six studies employed a mixed methods approach, followed by qualitative (*n* = 5) and quantitative designs (*n* = 4). The majority of studies (11 of 15) focused solely or in part on cities in the U.S., whereas three studies included cities in Europe. Among studies of U.S. cities, five focused on two significant greenway projects that have fostered green gentrification: Chicago’s 606 [25,35,36] and the Atlanta BeltLine [37,38].

Table 2 shows the 15 studies organized by greening intervention (columns) and health outcomes/pathways (rows). Overall, the most studied greening interventions were new greenways or parks (*n* = 9 [25,27,35,36,37,38,39,40,41], whereas two focused on street greening [15,42] and three on environmental remediation, such as brownfield cleanup and redevelopment [27,42,43]. Two studies focused on more than one greening intervention, covering environmental remediation followed by either street greening [42] or the creation of new parks [27]. Among the 15 included studies, 12 focused on neighborhoods that experienced gentrification, at least in part, because of greening initiatives (i.e., green gentrification). Three studies, however, examined the impacts of green space on health or health pathways across a variety of gentrifying neighborhoods that might not have gentrified as a direct result of new greening interventions [44,45,46].

The most studied health outcome or pathway was park/greenway use and physical activity (*n* = 8), followed by sense of community or belonging (*n* = 5), sense of safety (*n* = 4), and self-reported health and well-being (*n* = 2; see Table 2). Two other health pathways include the loss of informal green spaces that were used to grow food [41] and change in air pollution due to freeway rerouting [42]. As for greening interventions, several studies (*n* = 6) examined more than one health outcome or pathway [25,35,36,38,40,45] (see Table 2). More details about greening interventions and health outcomes or pathways are reported in Table 1.

In the sub-sections below, we report our synthesis of the most significant findings of the 15 included studies regarding the relationships between greening initiatives and health in the context of gentrifying neighborhoods. We organized the most salient takeaways from our synthesis into five sub-sections that enabled us to map out the current state of the evidence on green gentrification and health. The content of the five sections reflects topical areas that are evident in the rows of Table 2 (e.g., Section 3.3 focuses on sense of safety in green space).

### 3.2. Green Space Use and Physical Activity

Findings on whether green space use/physical activity differed by demographic characteristics were mixed. Among the eight studies on green space use/physical activity (see Table 2), four examined differences based on race/ethnicity [25,35,37,45], and two others between neighborhoods at different stages of gentrification [38,40]. Findings on how green space use/physical activities varied by race/ethnicity were inconsistent, although more studies found that marginalized people used green space less than White people than studies finding the opposite. In a study of Atlanta (Georgia) and San Antonio (Texas), White people were overrepresented as greenway users compared to surrounding areas, whereas Black and Latino/a people were underrepresented [37]. Similarly, a study in Philadelphia showed that White people perceived a gentrifying neighborhood and its green spaces as more supportive of physical activity than long-time BIPOC residents [45]. But in an investigation of Chicago’s 606 trail (Illinois), Latinos/as were more likely than other racial/ethnic groups to use the trail more frequently, to be motivated to do so for health reasons, and to have higher physical activity as a result [25]. And another study on the 606 showed that trail use was segregated by race/ethnicity [35]: Latino/a people gravitated around the western end of the trail (surrounded by majority-Latino/a areas), and White people were mostly using the eastern side of the trail (surrounded by majority-White neighborhoods). These findings about segregated use were also highlighted in Kraft et al.’s [25] work.

Findings about whether green space use differed based on stages of neighborhood gentrification suggest that green space use is lower in places where longtime, low-income residents are at a higher risk of displacement. In a study in Barcelona (Spain), Oscilowicz et al. [40] found that green spaces were used more frequently in a neighborhood at the early stages of gentrification (where residents had more access to affordable housing) than in a late-stage gentrification area (with more displacement pressures due to tourism development). Palardy et al.’s [38] study of Atlanta showed that residents of a White affluent neighborhood used the BeltLine at higher rates than those of a gentrifying, majority-Black neighborhood.

One study in Birmingham (Alabama), focused on green space use and physical activity without examining differences by demographic groups. The authors found that Black residents felt that their neighborhood provided more support for physical activity after public housing was redeveloped into mixed-income housing with ample green space and walking paths [39]. The positive impact of new green spaces might be explained, at least in part, by the fact that longtime, low-income Black residents had access to affordable subsidized housing through the HOPE IV program, and therefore might not fear displacement as residents not living in subsidized housing.

### 3.3. Green Space Safety

Multiple studies reported that green space safety is an important issue that might affect the use of green space (see Table 2). In most circumstances, perceptions of unsafe parks or greenways were associated with lower use of green spaces. In Chicago, Harris et al. [36] found that White greenway users perceived the 606 as less safe in the majority-Latino/a area that is currently gentrifying, likely due to stigma. Perhaps as a result, White people rarely used the western side of the trail, surrounded by such gentrifying areas [35]. Similarly, fears of crime related to tourist activities contributed to lower green space use in a late-stage gentrification neighborhood of Barcelona [40]. Along these lines, a study in Philadelphia (Pennsylvania) showed that longtime Black residents perceived green spaces as less safe than White gentrifiers, and fear of violent crimes created barriers to physical activity in parks for Black residents, including barriers to children’s play [45]. Somewhat surprisingly, another study about the 606 reported that Latinos/as were more likely than other racial/ethnic groups to be concerned about safety while on the 606, but they used the trail more frequently than other groups [25]. This divergent finding might be because Latinos/as living near the 606 perceive the trail as a safer space than other nearby parks [35], and thus might decide to use the 606 even if they do have some safety concerns about it.

### 3.4. Sense of Community and Belonging

Findings on how green gentrification affected sense of community and sense of belonging were identified in five studies [15,35,38,43,46] (see Table 2). The findings of these studies are quite consistent: In most studies, marginalized residents feel that sense of community is lower after green gentrification occurred, or that they expressed a diminished sense of belonging in green spaces and their neighborhoods more broadly. In particular, Twigge-Molecey’s [46] work in Montréal (Quebec, Canada), showed how fewer options for affordable housing strained the neighborhood’s social networks, and how longtime residents felt disenfranchised by the fact that parks were only improved after gentrifiers started to move to the area.

Changes to the social environment that accompany green gentrification also relate to different dimensions of health and well-being. For instance, Harris et al. [35] describe how green gentrification along Chicago’s 606 trail is associated with concerns of discrimination, conflicts on the trails, and social exclusion, particularly amongst Latino/a users who tended to stay on the western part of the trail. Such feelings of exclusion from new green spaces (or part of them) might result in lower rates of visitation among longtime, low-income BIPOC residents (e.g., [35]). In another study in Ghent, Belgium, longtime residents noted that green gentrification can change the community power dynamics in ways where they are excluded and devalued in the ‘place making’ process [15]. Such challenges to the social dynamics and sense of community can also influence how some people perceive the actual green spaces. For example, a study of residents along the Atlanta BeltLine found that wealthier White residents tended to feel a greater sense of connection and ‘psychosocial empowerment’ as it relates to its trail system in comparison to Black gentrifying areas [38]. Also, as some longtime residents develop resentment toward gentrifiers, they note an atmosphere of segregation or self-segregation in a community that has strained social interactions [15]. Similarly, residents of another study describe how green gentrification can threaten their neighborhood’s sense of community, displace residents and local businesses, and attract gentrifiers who are less attached to the neighborhood [43].

### 3.5. Self-Reported Health and Well-Being

Two studies in our sample focused on how green space had differential impacts on health and well-being based on socioeconomic status (see Table 2). While greater exposure to green space can significantly lower the odds of poor health for residents of gentrifying neighborhoods, a study in New York found that only residents with a higher level of education and income appeared to benefit from this resource [44]. In a study focusing on Bangalore, India, respondents mentioned that the creation of parks allowed them to have walking paths, aesthetic improvements, and areas for children to recreate [27]. The increase in park access was linked with higher happiness and self-reported well-being for the wealthiest and middle-income residents [27]. However, the two-sided coin of green gentrification can lead to mixed results when it comes to health and well-being. For example, while Bangalore enhanced its provision of cultural ecosystem services, the feeling of being unwelcome, unfamiliar with urban nature, or having fewer options to adapt to ecosystem changes (due to lack of financial resources and stable housing) left some urban poor residents further marginalized [27].

### 3.6. Other Pathways to Health

The included papers discussed two additional pathways to health in relation to green gentrification (see Table 2): reduced access to lands used for food production and other community needs (e.g., nature-based recreation and ecological preservation) and improvements in air quality [41,42]. Specifically, Anguelovski et al. [41] found that the planning of a Green Belt in Medellin (Colombia) led to community concerns about lack of meaningful engagement, rendering the community’s existing socio-ecological relationships with land invisible. As a result, community access to lands used for food production was negatively affected, and their existing sustainable land use practices, farming community practices, and food networks were ignored. On a different note, Patterson and Harley [42] showed that the re-routing of a freeway in West Oakland (California) and the construction of a street-level boulevard with trees and other greenery had positive impacts on air quality, specifically, concentrations of nitrogen oxides and black carbon. Although air pollution decreased, these projects likely spurred green gentrification and a greater decrease in Black populations along the green boulevard than in West Oakland as a whole. The availability of affordable housing along the boulevard, however, helped several low-income residents remain in the neighborhood.

Fears of displacement and a decrease in affordable housing were other major pathways discussed in some articles identified in this scoping review (e.g., [15,40,42]). As housing is a key social determinant of health [47], concerns that green gentrification will diminish options for residents to remain or have access to quality housing options contradicts the vision for health equity. The combination of diminished housing options, fragmented social networks, and psychological distress can exacerbate environmental health disparities.

### 3.7. Connecting the Main Findings: A Literature Map

**Figure 2:**
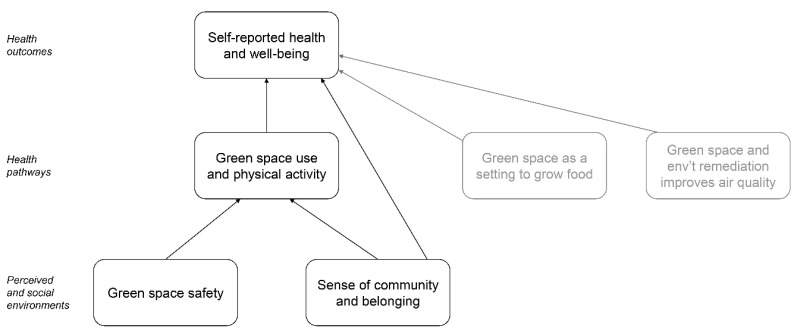
Literature map based on the 15 studies identified in this review. Text in gray represents content included in fewer studies.

Several of the research areas described through our main findings (Section 3.2, Section 3.3, Section 3.4, Section 3.5 and Section 3.6) have clear connections established (see Figure 2). Specifically, the research areas are represented through rectangles, whereas their connections are depicted through arrows. We established that those connections existed either because they were evident in the 15 studies included in our review or because they have been established in the literature about green space and health. For example, articles in our review showed connections between green space safety and use [36,40,45] and between sense of belonging and green space use [35]. Also, numerous other studies link higher green space use to better health outcomes as well as green space improving air quality, which in turn leads to better health [31,48]. We also organized the research areas we identified into three levels. On the lower part of the map, we included elements of the perceived and social environment, which are social determinants of health listed in ecological models of health [49]. In the middle of the map, we depicted health pathways such as physical activity, while health outcomes are represented at the top of the map and include self-reported health.

Some of the connections among the main research areas can be further clarified. Specifically, concerns that green gentrification can fragment an area’s social network and diminish its sense of community [43] which also relates to subjective well-being. The dynamics between gentrifiers and long-time residents can lead to tension and a strained social environment in some cases, as long-time residents might feel unwelcomed or that they no longer belong in their neighborhood [46]. Also, as green gentrification can limit options for affordable housing and retail outlets [46], the opportunity to benefit from green spaces is often short-lived for many low-income, long-time residents. Not only do the decreased options in affordable housing due to green gentrification limit access to quality green spaces, but because housing is a social determinant of health, shrinking affordable housing options negatively affect multiple dimensions of health and well-being [11,12]. Among the studies included in one review, one found that new green spaces had positive impacts on long-time, low-income BIPOC residents’ physical activity in the context of a mixed-income community, where such residents had access to affordable housing [39]. Finally, although increased availability of green spaces may promote physical activity, underlying social tensions can worsen the experiences of BIPOC communities in green spaces located in gentrifying neighborhoods [35].

## 4. Discussion

Motivated by the need to address health disparities, public health practitioners and urban planners have advocated for greening interventions, such as the creation of new parks, in low-income minoritized communities [21,50]. But in many cases, such greening interventions have backfired and resulted in “green gentrification,” describing the arrival of wealthier, and often White, newcomers to marginalized neighborhoods and increases in housing prices, which can contribute to displacing the lowest-income residents [14]. In this scoping review, we identified 15 peer-reviewed journal articles that speak about the complex interrelationships between gentrification, green space, and health. The results revealed insights that align with previous systematic reviews on gentrification (in a general sense) and health (e.g., [8,9,10]), showing unique findings that relate to green gentrification, and demonstrate gaps in empirical evidence needed to advance urban health justice through urban greening [14]. The following sections expand on these three areas.

### 4.1. Relation to Previous Work on Gentrification and Health

Similar to previous reviews on gentrification and health (e.g., [8,9,10], we found that marginalized people such as low-income and racially/ethnically diverse populations are most often negatively affected by green gentrification. Specifically, Bhavsar et al. 2020 found that Black people and older individuals tend to have worse health impacts from gentrification than White and younger people, specifically as for physical health outcomes [8]. Several studies in our review showed divergent health impacts between marginalized and more privileged groups. For example, one reported that marginalized groups in gentrifying neighborhoods either had no positive impacts on self-reported health from green space proximity, whereas people of higher socioeconomic status reported protective effects [44]. Overall, our work confirms the results of previous work on gentrification and health showing that, when it comes to green gentrification, marginalized groups are most often the ones suffering the worst impacts on health and well-being.

### 4.2. Unique Findings on Green Gentrification and Health

Our review on green gentrification and health revealed unique findings tied to the differential benefits of green spaces in the context of gentrifying neighborhoods and to broader neighborhood transformations. Several studies included in our review focused on differentials rates of green space use and physical activity. Although the findings of those studies are somewhat mixed, more of them show that White residents use green space more frequently and are more physically active in them [37,45] than vice versa [25]. Studies comparing green space use by stage of neighborhood gentrification suggest that use may be higher in places where residents experience lower displacement pressures [38,40].

Other studies included in our review highlighted safety issues in green spaces, which in most circumstances contributed to lowering green space use [36,40,45]. Although one previous review on gentrification and health covered studies reporting changes in neighborhood crime [9], such studies did not specifically focus on safety issues in and around parks and greenways. In our review, we also find that feelings of green space safety often vary across racial/ethnic lines, with White people stigmatizing BIPOC individuals and communities in some circumstances [36]. However, on other occasions, BIPOC people experienced a lower sense of safety in green spaces than White people [25,45].

In addition, some studies in our review covered sense of community and sense of belonging in places undergoing green gentrification [15,35,43] or broader gentrification processes [46]. The results of such studies show quite consistently that marginalized residents perceive their sense of community to be lower after green gentrification occurred, and/or a lower sense of belonging in their neighborhoods and green spaces, which are often perceived as serving wealthier newcomers. As noted earlier, such feelings of not belonging in new green spaces might, in turn, result in longtime low-income BIPOC residents visiting green spaces less frequently (e.g., [35]) and therefore accruing fewer health benefits from such green spaces. Interestingly, sense of community and sense of belonging were not covered in the aforementioned previous reviews on gentrification and health. This might be due to narrower definitions of health used in those reviews compared to ours, as well as to our use of social sciences databases (e.g., JSTOR) in addition to medical and epidemiological databases (e.g., PubMed) for our search.

### 4.3. Literature Gaps on Green Gentrification and Health

Through our scoping review, we also found that the literature on green gentrification and health is relatively small but has been growing in the last three years. Our analysis showed significant literature gaps that open avenues for future research. First, mental health issues such as stress and depression are understudied in the context of green gentrification and the health implications of green space in gentrifying neighborhoods. Previously published research demonstrates that green space has positive impacts on mental health [51,52,53], but research has not examined whether gentrification affects that protective effect.

Second, we did not identify work that examined specific physical health outcomes that are associated with green space use in the context of green gentrification, such as cardiovascular markers (e.g., blood pressure), body mass index (BMI), and diabetes. Comparing these and other physical health outcomes between longtime, low-income residents and more affluent newcomers would inform whether and how green gentrification moderates relationships between active green space use and physical health [31,51]. Specifically, additional insight on both potential barriers to physical activity and enabling factors that facilitate the active use of green space in gentrifying neighborhoods based on length of residence and income level can be informative.

Third, we did not find studies that compared the health outcomes of low-income residents of gentrifying neighborhoods who stayed in place to those who were displaced by green gentrification. Understanding which groups are experiencing better health and knowing why can have significant implications for urban planning and public health practice. Specifically, if this evidence showed that people who stay in place fare better than those who are displaced, more investments would need to be made to preserve or create affordable housing in areas undergoing green gentrification [50]. Also, such evidence can inform the provision of health care services, public transportation, parks, and recreational programs in suburban neighborhoods, where many displaced people tend to relocate as indicated by increases in the shares of low-income and minoritized residents in these areas [54].

Fourth, and more broadly, our search did not yield studies that investigated the extent to which green gentrification results in displacement and the associated health impacts of being uprooted from one’s neighborhood. This seems like a particularly significant omission given the potentially negative health outcomes that could result from residential displacement (see [55]). Although it is difficult to measure displacement and know where displaced residents have relocated [6], insight on the potential health consequences of displacement from green gentrification can help public health and urban planners evaluate the pros and cons of greening initiatives in marginalized communities.

Fifth, although our review raises important questions about the inequitable experience of benefits from green interventions in gentrifying neighborhoods, understanding who benefits from greening interventions in gentrifying neighborhoods remains a critical question. This work would have important implications for environmental justice and health equity. While scholars have explored the role of urban green spaces to alleviate some health inequalities [56,57,58], the extent that these benefits manifest in gentrifying neighborhoods is not fully understood.

Sixth, more research is needed to understand “what worked” in effective greening initiatives in low-income BIPOC communities that improve health and well-being for longtime residents and minimize green gentrification. Specifically, research is warranted on how to successfully implement greening interventions that both improve health outcomes for marginalized communities and help them stay in place after greening interventions are completed. Projects like Washington DC’s 11th Street Bridge Park, which have been deemed as models to limit displacement [59] could be studied in depth to extend their best practices to other settings.

Finally, the influx of wealthier residents to a gentrifying neighborhood can shift social norms in ways that lead to racial tension and resentment among socioeconomic classes [60]. Other negative sentiments can also occur when gentrifiers become socially isolated from long-term residents [61]. Gentrification can raise suspicions among residents and long-term supporters of communities when ‘outsiders’ come to the area with a different vision. Likewise, gentrification and the subsequent changes in an area’s social fabric can result in underserved residents not feeling welcome [21]. Other concerns linked to green spaces in gentrifying neighborhoods include the demise of an inclusive atmosphere and sense of community where ‘place making’ includes the vision and endorsement of long-term residents [15].

### 4.4. Strengths and Limitations of This Review

The main strength of this review is its focus: To our knowledge, this is the first effort to map out the literature on green gentrification, health, health pathways, and well-being. Another strength is in our methodology, as we built on the Preferred Reporting Items for Systematic reviews and Meta-Analyses Extension for Scoping Reviews (PRISMA-ScR) guidance in combination with other established approaches for review papers. As an additional strength, we drafted a qualitative synthesis of the existing literature that enabled us to map out the existing macro areas of research in the growing field of green gentrification and health. As a final strength, we presented a comprehensive list of topics relevant to public health and urban planning that the literature on green gentrification and health has yet to explore (see Section 4.3).

Our review also has some limitations that can be addressed in future systematic evaluations of the evidence on green gentrification and health. Although we attempted to be as comprehensive as possible with our multi-step, multi-method search strategy, we may have missed some studies that were either not written in English or that used different terminology to describe gentrification or gentrifying neighborhoods than the terminology required in our inclusion criteria. Although the relatively small number of studies we identified (*n* = 15) can be considered a limitation of this study, we also think that such a small number is a finding in itself, and it is not uncommon for reviews focusing on nascent fields of study to find few relevant empirical articles on the topic [10,62,63,64,65].

We also did not assess the quality of studies included in our review nor did we assess for potential biases that could have impacted the results of said studies. Yet a consistent assessment of the quality of studies would have been difficult given the various methodologies used in the 15 articles we included in our scoping review; most instruments to evaluate possible methodological bias only apply to quantitative studies (see [66]). Finally, we did not include grey literature in our review, which might have over-represented studies reporting significant and positive results in our sample, which tend to have a better likelihood of being published in peer-reviewed journals than other research (i.e., publication bias). Recognizing that innovation from public health practice often originates in the field rather than from academic research, limiting our review to peer-reviewed publications might have left out relevant information for our research questions.

### 4.5. Implications for Public Health and Urban Planning

The main findings of our review can inform the work of public health professionals and urban planners who are interested in advancing health equity through greening initiatives. First, our findings about racial/ethnic disparities in green space visitation suggest the need for design features and recreation programs that encourage visits among marginalized residents of gentrifying neighborhoods. Specifically, research has suggested that parks and greenways in gentrifying communities are most often designed to meet the needs and aesthetic preferences of affluent White newcomers, as opposed to longtime low-income BIPOC residents [22]. To this extent, early, meaningful, and consistent engagement of residents of communities targeted for urban greening projects is critical to ensure that long-time residents have a voice in designing green spaces that they would like to use [50]. Along the same lines, recreation programs held in new or renovated green spaces located in gentrifying communities should be tailored to the cultural preferences, financial means, and everyday needs (e.g., child care) of diverse and low-income communities [50,67]. And public health officials could implement initiatives to encourage green space use among marginalized groups in communities undergoing green gentrification, such as those conducted by *promotores de salud* in Latino/a neighborhoods [68].

Second, creating inclusive park designs and recreational programming through meaningful community engagement might also help increase a sense of belonging in green spaces among longtime, low-income BIPOC residents. For example, a study in Philadelphia found that community engagement to design park improvement and programming was associated with community ownership of the renovated parks and with feelings that the park is an integral part of the social fabric of the community [67]. Similarly, the use of a *promotores de salud* model in Los Angeles resulted in higher community empowerment in park programming [68]. These examples show the importance of holistic approaches that integrate community-driven design, recreational programming, and health promotion efforts to ensure that marginalized communities visit and feel welcome in green spaces in gentrifying communities.

Third, the results of this review also show that the perception of unsafe green spaces can limit green space use and physical activity in gentrifying communities. BIPOC communities in the U.S. are well aware that asking law enforcement to help address safety issues in public spaces could be a double-edged sword. Despite the recent police killings of George Floyd, Breonna Taylor, and many other Black individuals and the ensuing growth of the Movement for Black Lives, racial profiling and violence targeting BIPOC people are still prevalent in the country [69]. And a research study in Chicago shows that BIPOC youth are often the object of community policing on the 606 trail, which has resulted in some youth limiting their visit to the trail [26]. What can planners and elected officials do, then, to make these spaces feel safer for BIPOC communities without relying on policing? One option is, again, providing meaningful recreational programming that promotes the positive use of parks, such as Los Angeles’ Summer Night Lights program, which activates parks during summer nights, when gang activity tends to be higher [70]. Further, efforts such as community-driven safety initiatives could also be implemented in neighborhoods undergoing green gentrification [71].

Fourth, the findings of at least two studies in our review suggest the importance of having access to affordable housing for marginalized people to reap the benefits of green space in gentrifying communities [39,40]. Creating and preserving affordable housing in places undergoing green gentrification is arguably one of the major challenges for equity advocates in these communities [72]. One of the reasons why doing so is challenging lies in the fragmentation between planning for housing and green space, wherein funding, organizational skills, and jurisdiction rarely align [22]. Thus, investments in urban greening projects must integrate displacement protections for current residents and affordable housing production. In this vein, learning from promising practices such as the creation of joint-development frameworks to promote the simultaneous development of parks and proximal affordable housing is key [73].

## 5. Conclusions

As gentrification involves a complex process of cultural, social and racial dynamics, its reach extends across several cities and neighborhoods around the world [74]. Among the different mechanisms that foster gentrification, in this scoping review, we focused on green gentrification and its impact on health, health pathways, and well-being. Green gentrification is a complex phenomenon as marginalized communities often face the conundrum of choosing between health-promoting green spaces and gentrification threats in their neighborhood [22,44]. Thus, the health consequences of green gentrification defy the premise of health equity which aims for all people to have the same opportunity to achieve optimal health. Insight on the environmental, economic, and social factors involved in changing cities is critical to support urban health for all [75]. As greening initiatives are often included in increasingly popular ‘smart cities’ initiatives [76,77], planners, public health officials, and policymakers need to pay particular attention to how green gentrification might affect health outcomes.

Through our scoping review, we uncovered a mixed picture of the impacts of green gentrification on health, health pathways, and well-being. We identified 15 articles published in countries around the world, with methodologies ranging from qualitative, mixed methods, and quantitative. One of the most consistent findings is that green gentrification contributes to a lower sense of community and sense of belonging among longtime, low-income BIPOC residents. We also found socioeconomic and racial/ethnic disparities in the associations between green space access and self-reported health, green space use, and physical activity. Long-time, low-income residents often reap fewer health benefits from new and renovated green spaces that wealthier and White newcomers to gentrifying neighborhoods. Further, issues of green space safety can hinder green space use especially for marginalized residents, and issues of stigmatizations also tend to negatively affect the green space experiences of BIPOC communities.

The risk of displacement associated with green gentrification reinforces the reality that access to new parks and green space is not enough for vulnerable populations since it is often temporary. Therefore, additional research on the policy tools (e.g., rent control, land trusts, housing cooperatives, social housing construction) that can combat displacement is also needed [78]. Interventions to combat gentrification should effectively avoid segregation and tactics of social containment [79]. As expressed by Rigolon and Nemeth, it is important to acknowledge that ‘*not all parks are created equal, and not all parks result in green gentrification in the same ways*’ ([17], p. 403). Enhancing our perspective on green gentrification and health can balance the conversations on urban development in ways that support health equity.

## Figures and Tables

**Table 1:** Characteristics and key findings of the 15 included studies

Citation	Methodology	Location	Study Population	Data Sources	Analytical Methods	Greening Intervention	Health, Pathways, or Well Being	Key Findings
Pearsall (2012) [43]	Qualitative	New York City, NY (USA)	42 residents of three neighborhoods undergoing environmental gentrification in New York City	Semi-structured interviews: Questions about residents’ perceptions of neighborhood change, such as adaptation to cost of living	Content analysis	Environmental remediation (i.e., brownfield development) in three neighborhoods of New York City	Sense of community	Residents felt that environmental gentrification threatened the neighborhood’s sense of community and character. With some long-time residents and businesses being displaced, residents felt that the newcomers were more transient and less committed to the neighborhood.
Twigge-Molecey (2014) [46]	Qualitative	Montreal, Canada: Saint Henri area	34 residents, particularly renters who have resided in the neighborhood for at least five years	Semi-structured interviews	Descriptive statistics and summary of observations through a four-fold typology	No intervention; gentrification occurring regardless of green space; The study explored gentrification and noted observations with its parks.	Sense of belonging; Access to new community groups and retail options	Affordable housing was absent and social networks were fractured; The restraints (or lack thereof) of dogs owned by affluent gentrifiers became a source of tension and cultural displacement in the neighborhood. While the tension added to the displacement pressure of some long-term residents, others welcomed the social contact and chance to meet affluent neighbors. Diverse perceptions of the park were reported. They were considered to improve the neighborhood yet seem to appear to accommodate affluent newcomers. Low-income residents noted fewer retail options with changes to the retail landscape.
Dulin-Keita et al. (2016) [39]	Mixed Methods	Birmingham, AL (USA): Tuxedo area	59 residents of HOPE VI projects; all African American and older than 18	Concept mapping: Five-step process to generate ideas, structure them, and analyze statements made by participants	First, statement analysis; then, “cluster formation, bridging values, cluster ratings, *t*-tests, and pattern match”	The HOPE VI development includes green space and parks	Physical activity	The creation of new parks removed some barriers to physical activity in the area (for children and adults). New parks were perceived to be safer than pre-existing play areas before the HOPE VI redevelopment (no syringes or broken glass). The new parks and walking paths had better lighting and were more aesthetically pleasing. Participants said that more Black males were running in the area and the police should know that it’s just an increase in physical activity (and should not harass them). Perceptions of drawbacks of this development were limited.
Derkzen et al. (2017) [27]	Mixed Methods	Bangalore (India): Seven urban lake communities	214 slum residents living in the study location	Household surveys administered through door-to-door interviews; Seven group mapping sessions with 3–6 long-term residents per session	Qualitative data analysis (mapping data) and descriptive statistics (survey data) are both assumed but not explicitly referenced in the article.	Environmental remediation through restoration of previously polluted artificial lakes; Creation of parks	Well-being through self-reported accounts of use of ecosystem services from urban lakes and ability to adapt to ecosystem changes	The very poor, who rely on the lakes for provisioning ecosystem services that promote human well-being have few options to adapt to the new circumstances associated with ecosystem changes (urbanization followed by restoration and subsequently gentrification). Changes in the ecosystem deprive the urban poor of both cultural and provisioning ecosystem services derived from the natural resources on which they depend. When stable housing and adequate financial resources to meet daily needs are coupled with green amenities, more low-income residents feel that green space has benefits for them.
Anguelovski et al. (2018) [41]	Mixed Methods	Barcelona (Spain), Medellin (Colombia), New Orleans, LA (USA)	Unspecified; the study did not enroll participants in all locations and focused on environmental characteristics	Secondary data from municipal records (Barcelona), interviews and participant observations (Medellin), and planning documents (New Orleans)	Regression and spatial analysis (Barcelona), qualitative analysis of semi-structured interviews and participant observation (Medellin), and planning document and project analysis (New Orleans)	New parks and gardens (Barcelona), new greenbelt created and informal green spaces removed (Medellin), and climate adaptation strategies (New Orleans)	Accessibility to land used for food production and other local needs and practices (social cohesion, nature-based recreation) affecting community well-being	In Barcelona, working-class ‘greened’ areas increased in the proportion of socially vulnerable residents who lived closer to green space, but in areas next to highways and areas with worse housing conditions. In Medellin, low-income residents were identified as losing access to vernacular green spaces and losing access to land used for fresh food production, on which their livelihoods often depend. In New Orleans, climate resilience planning efforts made the needs of BIPOC people invisible--ignoring the racialized and inequitable history of planning in the city and the disproportionate effects of flooding on these communities.
Keith et al. (2018) [37]	Quantitative	Atlanta, GA (USA) and San Antonio, TX (USA)	934 greenway users	Intercept surveys with greenway users	Descriptive statistics and ordinary least squares regression	New greenways: Atlanta BeltLine and Leon Creek Greenway in San Antonio	Greenway use, motivations for using the greenway, constraints to greenway use, and perceived benefits of the greenways	Whites were over-represented as users in both greenways compared to surrounding areas; Blacks and Latinos/as were underrepresented. Differences are particularly big in Atlanta for Black people, and the areas around the BeltLine were undergoing gentrification (in 2015). In both sites, Black visitors were more likely than White visitors to use the greenways to discover and experience nature; Latinos/as were more likely than White visitors to use the greenway to spend time with family and friends and to use the greenway for transportation purposes. Lack of free time was less a barrier for visitation among Black people (compared to Whites). Fear of crime was a stronger constraint for Asians/Others than for Whites. Blacks perceived that the trail was less difficult to access than Whites. “Cultural benefits were likely to be cited by Hispanic and Asian/Other visitors. Hispanics were also more likely to cite environmental benefits than any other visitor group”
Kraft et al. (2018) [25]	Mixed Methods	Chicago, IL (USA)	Includes a total of 602 study participants	Surveys were collected throughout the trail for multiple years	Descriptive statistics and multivariable regression models	New greenway developed (The 606)	Perceptions of trail use, sense of safety, motivations for use, and impact on physical activity	Many Latino/a trail users noted the frequent use of the trail, however, concerns around safety and possible exclusion were mentioned. Nearly half of respondents noted an increase in physical activity. Authors note patterns of trail use that are racially segregated.
Palardy et al. (2018) [38]	Quantitative	Atlanta, GA (USA)	418 residents living near two segments of the Atlanta BeltLine: Northside (affluent and White), and Southwest (gentrifying and majority-Black)	Survey of residents with systematic random sampling	Confirmatory factor analysis, independent samples *t*-tests, and structural equation modeling	New greenway developed (Atlanta BeltLine)	Greenway use, sense of connection to one’s neighborhood, sense of community	A larger share of residents in the wealthy White area supported the BeltLine, used the trail, and had higher psychosocial empowerment compared to the majority-Black gentrifying area. The BeltLine makes residents of the wealthier White area feel more connected to their neighborhood than the residents of the Black, gentrifying area. No differences in how the BeltLine fosters a sense of community. Residents of the White, affluent area feel that the BeltLine’s benefits outweigh its negative impacts more often than those in the Black gentrifying area.
Cole et al. (2019) [44]	Quantitative	New York City, NY (USA)	44,167 New York City residents (age 18 and older)	Individual-level health and demographic data (New York City); green spatial data from (New York City); and gentrification measures from (U.S. Census Bureau)	Logistic regression modeling	No intervention; Gentrification occurring regardless of green space Green spaces mentioned were parks in the neighborhood.	Self-reported general health	Greater exposure to active green space was significantly associated with lower odds of self-reporting of fair or poor health, for those living in gentrifying neighborhoods. Only those with high education or high incomes, within these neighborhoods, benefited from neighborhood active green space. A positive effect of active green space on general health was also found for non-Hispanic Whites and for those with higher levels of education independent of neighborhood gentrification status, but not for any other racial/ethnic groups or for those with lower levels of education.
Harris et al. (2019) [35]	Mixed Methods	Chicago, IL (USA)	Observed greenway users and 54 interviewees in Logan Square and Humboldt Park	Systematic observations (SOPARC); Interviews	Regression models; Thematic interviews	New greenway developed (The 606)	Sense of community; quality of social interactions; greenway use	Latino/a users expressed concerns about gentrification, discrimination, and exclusion, and they tend to stay on the western side of the trail. Five areas from the thematic analysis: community benefits, trail conflicts, social exclusion, environmental gentrification, and Latino/a resistance.
Patterson & Harley (2019) [42]	Quantitative	Oakland, CA (USA): West Oakland area	Unspecified; the study did not enroll participants and focused on environmental characteristics	Demographic data from the U.S. Census Bureau; traffic count data from the City of Oakland	Spatial analysis	Freeway rerouting (environmental remediation) and building a street-level boulevard (with trees and other greenery)	Vehicle emissions and near roadway pollutant concentration (NOx and black carbon)	Evidence of environmentally driven neighborhood change was identified based on larger decreases in the long-time Black population (−28%) and increases in property values (184%) in study areas compared to the neighborhood as a whole.
Schroeder et al. (2019) [45]	Qualitative	Philadelphia, PA (USA): West Philadelphia area	19 residents of a gentrifying neighborhood	Intercept interviews (on streets) and visual documentation of neighborhoods. Interview questions about physical activity and health promotion	Qualitative analysis: transcription of interviews, coding, creation of themes based on the codes (until data saturation was achieved)	No intervention; Gentrification occurring regardless of green space; Green spaces mentioned were parks in the neighborhood. A new park and running trail was built before the area started to gentrify	Physical activity, sense of safety	White residents (mostly newcomers) feel that the neighborhood supports physical activity. These people mentioned resources that were created when the neighborhood started to gentrify. Those included a new park and a new running trail (located in a nearby area). Black residents perceived barriers to physical activity, such as poorly maintained parks and lack of or unaffordability of recreation facilities (e.g., gyms). Black residents reported concerns about illicit substance use, poor relations with the police, gun violence and other violent crimes, which limited park use and physical activity. Blacks did not see the area as health-promoting.
Goossens et al. (2020) [15]	Qualitative	Ghent, (Belgium)	Study participants were recruited from Facebook group on green initiatives; *n* = 37 respondents	Semi-structured interviews of both gentrifying and longtime residents	Qualitative analysis: interviews were analyzed in Nvivo 10	Living Street Project (integration of street trees)	Sense of community; social interactions	Interviews showed concerns about residents’ ability to stay in the neighborhood. Gentrifying residents valued greening projects linked to urban renewal efforts. Gentrifiers also participate in place-making activities attached to the Living Street Project. Longtime residents express the project changed the essence and inclusive atmosphere of the area. Respondents note behaviors reflecting segregation or self-segregation; project resentment by some longtime residents
Harris et al. (2020) [36]	Qualitative	Chicago, IL (USA)	Users of the 606 trails and nearby residents; total of 86 study participants	Open-ended interviews and informal observations	Qualitative/thematic analysis to analyze observations and interviews	New greenway developed (The 606)	Sense of safety on the trail, greenway use	Overarching themes related to stigmas attached to Humboldt Park, trail aesthetics, and stereotyping of Latino/a youth. Efforts to overcome stigma through gentrification and resistance depending on location.
Oscilowicz et al. (2020) [40]	Mixed Methods	Barcelona (Spain)	173 parents and caretakers of primary school children living in two gentrifying neighborhoods	Observation of green spaces, survey with parents and caretakers of primary school children, and interviews with parents and caretakers	Descriptive statistics (observations), mixed-effects logistic regression (survey), and thematic analysis using both deductive and inductive codes (interviews)	Parks, squares with vegetation, and playgrounds. All green spaces can be used as play spaces by children. Some green spaces were recently built.	Use and perceptions of green spaces (for families and children, with a focus on play), sense of safety	In the area at a later gentrification stage, families and children use green spaces less and are less satisfied with them. In this area, there are more crime issues (related to tourism) and a lower sense of security. Green spaces were seen as serving tourists (as opposed to residents) to some extent. This might lead families and children to stay home (sense of insecurity). Families also feel the pressure of residential displacement. In the area at the early stages of gentrification, there is a higher use of green space and more place attachment. But residents are concerned that they might be displaced by increased housing prices: “short-term green benefits but possible long-term losses”

**Table 2:** The 15 include studies categorized by greening intervention and health outcome/pathway.

		Greening Interventions (or Exposures *)		
		Greenways and Parks	Street Greening	Environmental Remediation
Health outcomes and pathways	Green space use and physical activity	Dulin-Keita et al. (2016) [39]; Keith et al. (2018) [37]; Kraft et al. (2018) [25]; Palardy et al. (2018) [38]; Harris et al. (2019) [35]; Schroeder et al. (2019) * [45]; Harris et al. (2020) [36]; Oscilowicz et al. (2020) [40]		
	Sense of safety	Kraft et al. (2018) [25]; Schroeder et al. (2019) * [45]; Harris et al. (2020) [36]; Oscilowicz et al. (2020) [40]		
	Sense of community and belonging	Twigge-Molecey (2014) * [46]; Palardy et al. (2018) [38]; Harris et al. (2019) [35]	Goossens et al. (2020) [15]	Pearsall (2012) [43]
	Self-reported health and well-being	Derkzen et al. (2017) [27]; Cole et al. (2019) * [44]		Derkzen et al. (2017) [27]
	Other pathways	Anguelovski et al. (2018) ^a^ [41]	Patterson & Harley (2019) ^b^ [42]	Patterson & Harley (2019) ^b^ [42]

Notes: Some studies appear in more than one cell because they considered more than one health outcome or pathway or more than one greening intervention. *: Denotes study where gentrification was occurring regardless of greening interventions. ^a^: Loss of places to grow food; ^b^: Change in air pollution (NOx and black carbon).

## Supplementary Materials

The following are available online at https://www.mdpi.com/1660-4601/18/3/907/s1. Table S1: PRISMA Extension for Scoping Reviews Checklist, Table S2: Search Strategy Elements.
